# Serum Levels of Vascular Endothelial Growth Factor, Platelet Activating Factor and Eosinophil-Derived Neurotoxin in Chronic Spontaneous Urticaria—A Pilot Study in Adult Patients

**DOI:** 10.3390/ijms23179631

**Published:** 2022-08-25

**Authors:** Krzysztof Gomułka, Wojciech Mędrala

**Affiliations:** Clinical Department of Internal Medicine, Pneumology and Allergology, Wroclaw Medical University, 50-369 Wroclaw, Poland

**Keywords:** chronic spontaneous urticaria, vascular endothelial growth factor, platelet activating factor, eosinophil-derived neurotoxin, small molecules, skin

## Abstract

Chronic spontaneous urticaria (CSU) is a skin disease characterized by the presence of wheals, angioedema, or both for at least 6 weeks. Although, CSU is often regarded as autoimmune in nature, its etiology is not fully explained and interactions between various small molecules are still taken under account. The aim of this research was to investigate the mean serum concentration of vascular endothelial growth factor (VEGF), platelet activating factor (PAF), and eosinophil-derived neurotoxin (EDN) in relation to the disease activity and pruritus intensity in adult patients with CSU. Fifteen patients with CSU and 15 healthy subjects participated in this pilot study. Blood samples were taken to examine the mean serum levels of VEGF, PAF, and EDN by the enzyme-linked immunosorbent assay test (ELISA). The Urticaria Activity Score (UAS7) and The Visual Analogue Scale (VAS) were used to assess the disease activity and the pruritus intensity, respectively. Obtained results revealed that VEGF, PAF, and EDN concentrations were higher in patients with CSU compared with those of the control group, but only for VEGF it was statistically significant (*p* = 0.008). However, levels of all investigated cytokines were not significantly correlated neither with the disease activity nor with the pruritus intensity. Our results showed higher serum levels of VEGF, PAF, and EDN among CSU patients which may highlight a functional role of these cytokines in the disease’s pathogenesis. In contrast, VEGF, PAF, or EDN might not be useful to reflect the severity of symptoms.

## 1. Introduction

Chronic spontaneous urticaria (CSU) is a skin disease characterized by the presence of wheals, angioedema, or both for at least 6 weeks with the assumption that it occurs daily or close to it. The mainstay in the proper and effective treatment is still the use of antihistamines and in some cases short-term systemic corticosteroid. However, the use of omalizumab (recombinant DNA-derived humanized IgG1k monoclonal antibody that specifically binds to free human immunoglobulin E) has been a breakthrough for the patients with CSU [1,2]. The multifactorial and complex etiology of CSU is still not fully known and increasing knowledge on the pathogenesis has revealed target molecules in plasma (e.g., IL-4, IL-13, IL-5, IL-17, autoantibodies) and cells (e.g., infiltration of CD4+ T lymphocytes, monocytes, neutrophils, eosinophils, basophils) that could potentially be important during disease [3,4,5].

Vascular endothelial growth factor (VEGF-A, referred to as VEGF) is a heparin-binding homodimer glycoprotein that plays an important role in the angiogenesis process and is produced mainly by neutrophils, platelets, epithelial cells, and macrophages. Changes in the synthesis of VEGF may be associated with the pathomechanism of diseases such as: age-related macular degeneration, cancers and metastasis, ischemic heart disease, rheumatoid diseases, chronic obstructive pulmonary disease, or asthma [6,7,8].

The platelet activating factor (PAF, 1-O-alkyl-2-acetyl-sn-glycero-3-phosphocholine) is a bioactive phospholipid that has been found to be produced by basophils, eosinophils, mast cells, lymphocytes, macrophages, as well as platelets and endothelial cells. PAF binds to its specific receptor (PAFR) expressed on, i.e., mast cells and basophils. Degradation of PAF is regulated by the enzyme PAF-acetylhydrolase (PAF-AH). PAF is involved in the pathogenesis of cardiovascular diseases, psoriasis, sepsis, and allergic diseases like asthma, allergic rhinitis, anaphylaxis, and urticaria [9,10,11]. Rupatadine is a long-acting H1-antihistamine that antagonizes PAF and has been shown to be effective to alleviate symptoms of urticaria and allergic rhinoconjunctivitis [12].

Eosinophil-derived neurotoxin (EDN) is an RNAse found in eosinophil granules that has cytotoxic properties, can reduce activity of viruses and bacteria, has a neurotoxic effect, and induces neutrophil and monocyte chemotaxis. High level of EDN may have detrimental effects on the surrounding tissues being connected with the overproduction of matrix metalloproteinase-9 and enhances local remodeling in eosinophilic chronic inflammation. EDN has been shown to be implicated in the pathophysiology of, i.e., asthma, where it correlates with disease severity and with airway hyperreactivity [13,14,15].

The aim of our study was to assess the mean serum concentration of VEGF, PAF, and EDN in adult patients with chronic spontaneous urticaria in comparison with healthy controls. These three molecules were selected by considering a review of the available literature and still a small amount of work on this subject. Furthermore, the obtained results were used to verify possible correlations between the abovementioned molecules’ levels and disease activity and the degree of itching intensity assessed subjectively.

## 2. Results

**Figure 1:**
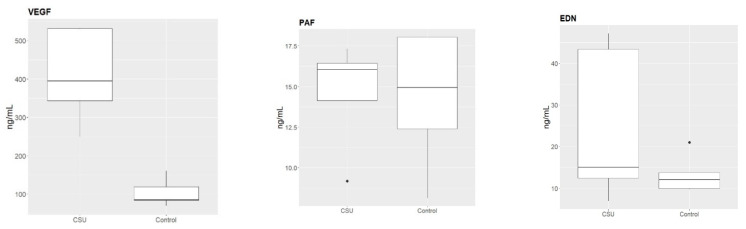
The mean serum concentrations of the examined molecules in patients with chronic spontaneous urticaria (CSU) and healthy control.

The studied sample showed that the mean VEGF serum concentration was higher in patients with CSU than in the control group, and this result was statistically significant (*p* = 0.008). Moreover, the mean PAF and EDN serum levels were also higher in patients with CSU compared to those in the controls, but differences were not statistically relevant (*p* = 0.999, *p* = 0.421, respectively). In patients with CSU, correlations between the biomarkers’ concentrations were VEGF vs. PAF: r = 0.70, VEGF vs. EDN: r = −0.30, PAF vs. EDN: r = −0.30. In the control group, the correlations were VEGF vs. PAF: r = 0.46, VEGF vs. EDN: r = −0.40, PAF vs. EDN: r = −0.05. The values and differences in serum concentrations of examined molecules in CSU patients and the control group are shown on Figure 1 and in Table 1.

Additionally, there was no significant correlation between neither VEGF nor PAF or EDN concentration and activity of CSU measured with UAS7 scale. On the other hand, pruritus was observed in 100% of patients with CSU and its average intensity assessed using VAS expressed moderate severity of itch. Nevertheless, the mean serum VEGF, PAF, and EDN concentrations did not correlate with the intensity of the itch sensations in the examined patients. The data about the disease’s severity in the examined patients with CSU are shown in Table 2.

## 3. Discussion

The incidence of CSU is growing worldwide, affecting the global population. Symptoms of CSU are unpredictable in terms of both course and duration and may persist for many years in many patients. Since CSU skin lesions (the wheals with well-circumscribed non-pitting edema and blanched centers, usually surrounded by erythema) are visible and accompanied by itching, it makes a significant impact on the patients’ quality of life. Females tend to be more affected by urticaria than males, and the disease seems more common among adults than among children and the average prevalence for lifetime of CSU risk is 1.4%. Therefore, a better understanding of its highly heterogeneous pathogenesis, which affects intracellular signaling defects and autoimmune processes, is needed [16,17,18].

In our study, to gain better understanding of the role of cytokines in immunopathogenesis of CSU, we focused on VEGF, PAF, and EDN—the main objectives were to signify the serum level of these molecules in our patient cohort with CSU in comparison to healthy controls and moreover to assess its possible correlation with the activity of disease. The findings of our pilot study showed higher mean serum VEGF, PAF, and EDN concentrations in examined urticarial patients compared with healthy individuals. It may be assumed that VEGF, PAF, and EDN plays an important role in the complex pathomechanism observed in patients with CSU. In available literature, bioactive mediators like various leukotrienes, prostaglandins, cytokines, or lipids are “regulators” of allergic disease pathophysiology in the development not only of CSU, but also asthma, allergic rhinitis, atopic dermatitis, food allergy, and anaphylaxis. Their role is crucial in cellular mechanisms including signaling cascades, chemotaxis, or cells degranulation [19,20,21]. Besides, a role of cells like basophils and eosinophils in the pathology of skin disorder has been described, indicating that some mediators are synthesized and released during CSU. Molecules, among VEGF, PAF, and EDN, have been demonstrated in dermatitis skin lesions, which might have an effect on the surrounding tissue, be strong modulators of the migratory response, and reflect the „priming” of eosinophils and basophils [22,23,24].

According to data from the literature, CSU might be associated with the increased numbers of new vessels and inflammatory cells, and elevated vascular marker levels have also been described [25,26,27]. A recent study by Zhao et al. [28] found that the sera of CSU patients induce mast cell production of VEGF via the PI3K/Akt/p38 MAPK/HIF-1 α signaling pathway and eosinophils produce VEGF, histamine, and other inflammatory mediators. It agrees with our findings that confirm overproduction of VEGF in patients with CSU but there was no remarkable correlation between plasma VEGF concentration and disease activity and pruritus severity. Nevertheless, this molecule could be regarded as a potentially important mediator and new possible therapeutic pathway in patients with CSU.

Some studies have indicated that in the group of lipid mediators PAF might have a role in the immunopathogenesis of CSU. Interestingly, work by Ulambayar et al. [29] showed significant increases in serum PAF levels and decreases in PAF-AH especially in patients with CSU refractory to antihistamines compared with healthy controls. Moreover, findings of Jenks et al. [30] with intradermal injection of PAF has been found to induce transient wheals with increases in vascular permeability followed by neutrophil infiltration. Additionally, other authors have suggested perivascular cellular infiltration of neutrophils, eosinophils, T cells, and monocytes, as well as increased expression of PAF, IL-6, and TNF-α in urticarial lesions [31]. Taken together, our present findings, showing that PAF concentration was higher in patients with CSU than in the control group, and previous reports from the literature in this matter, suggest an important role of PAF in the pathogenesis of the disease.

Eosinophils, as major effector cells in the inflammatory response by perivascular infiltration and releasing granule proteins (e.g., major basic protein, eosinophil cationic protein, eosinophil peroxidase, or EDN), have been implicated in the pathogenesis of many diseases, among others atopic dermatitis, urticaria, and asthma [32,33,34]. Our results are in accordance with the study by Saleh et al. [35], who found that the mean EDN serum levels were higher in patients with CU than the control group. Moreover, Saleh et al. revealed a positive correlation between serum EDN levels and disease severity, which was not found in our study—this may be due to the different number of participants in both studies, and different number of days considered on the UAS scale (four days in the study by Saleh et al., seven days in our study). The above data seem to confirm EDN role in the pathogenesis of the disease and suggest its usefulness as a part of the diagnostic and prognostic approach to CSU patients in the future.

In our study, VEGF, PAF, and EDN levels were different in the patient cohort than in the healthy control group, which might indicate the cytokine imbalance as being a part of the pathogenesis of CSU. However, it has been unclear if the examined molecules are related only to chronic inflammation in CSU or whether they relate to specific biochemical reactions, e.g., within the skin. Moreover, our obtained results have failed to find the correlation between these molecules’ levels and itching, which is an important subjective symptom for CSU patients. The major limitation of our pilot study is the small group of patients included with no relations between other biochemical markers and general inflammation that were investigated, as well as the absence of a positive control group, such as atopic dermatitis or psoriasis as an inflammatory dermatosis. In our opinion, a larger scale investigation should be performed for further confirmation.

## 4. Materials and Methods

### 4.1. Examined Groups

This pilot study included a total number of 30 participants, 6 males and 24 females, aged from 21 to 56 years (36.91 ± 10.21 average age). The study was approved by the local ethical committee (protocol code KB-224/2020) and all participants signed informed consent. The patient sample was composed of 15 subjects, 3 males and 12 females, aged from 30 to 56 years (43.2 ± 10.62 average age) who had a diagnosis of CSU established based on the precise medical history and complex physical examination before participation in our study. The control group consisted of 15 subjects, 3 males and 12 females, aged from 21 to 40 years (31.67 ± 6.77 average age) that were healthy non-atopic volunteers with negative medical history toward chronic skin diseases. The exclusion criteria for patients with CSU and healthy controls included: lack of consent, age under 18 or over 70 years, presence of cutaneous inflammatory disorders, significant comorbidities, pregnant and lactating females, taking systemic corticosteroids or long-acting antihistamines (up to 14 days prior to the study).

### 4.2. Blood Collection and Biochemical Analysis

From all patients with CSU and healthy controls, 5 mL of venous blood sample was taken to the coagulation activator tube (Saerstedt AG & Co., Nümbrecht, Germany) and centrifuged at room temperature at 1700 rpm for 16 min. In the next step, the serum was separated, transferred to the Eppendorf tube (AG, Hamburg, Germany) and kept frozen at −70 °C until further analysis. The mean serum concentrations of VEGF, PAF, and EDN were measured once, all in one plate, with the human enzyme-linked immunosorbent assay (ELISA) kits, following manufacturers’ instructions (Wuhan EIAAB Science Co., Ltd., Darmstadt, Germany). This ELISA detects human VEGF, PAF, and EDN with a minimum detection limit of 9.0 pg/mL, 0.12 ng/mL, and 6.0 ng/mL, respectively. To determine the concentrations, 4-parameter logistic (4-PL) curve-fit was used.

### 4.3. Disease’s Severity

The Urticaria Activity Score (UAS) was used for the assessment of disease activity in patients with CSU. This questionnaire analyses the number of wheals and the intensity of pruritus in a recommended 7-day monitoring period (UAS7), with once-daily documentation (resulting in a summary score from 0 to 42 points) [36]. Additionally, intensity of itch was evaluated with the Visual Analogue Scale (VAS) as an average pruritus from the previous week. Patients were asked to assess the intensity of the itch, where 0 points mean no itching, and 10 points mean the worst imaginable itch. To accurately interpret the results, they were ranked as follows: 0–2.9 points—mild pruritus, 3–6.9 points—moderate pruritus, 7–8.9 points—severe pruritus and 9–10 points—very severe pruritus [37].

### 4.4. Statistical Analysis

In statistical analyses, the Bartlett test, the *U* Mann–Whitney test, and the χ^2^ test were used. The Spearman correlation coefficient test was performed for analysis of selected parameter pairs. Statistical analysis was performed using the Statistica 13.3 software. *p*-values less than 0.05 were considered statistically significant.

## 5. Conclusions

The presented pilot study is one of the reports simultaneously showing an increased serum concentration of VEGF, PAF, and EDN in patients with CSU, in which the VEGF level was significantly higher compared to the control group. In contrast, none of the investigated molecules were relevant to disease activity by UAS7 and itch intensity by VAS. Our findings may be useful in the investigations of pathophysiology of CSU, which is a disease of complex, multi-pronged, and intertwined mechanisms. We believe that more studies on a larger cohort of patients than the one investigated in the present study may be conducted to clarify whether VEGF, PAF, and EDN may be potential biomarkers of CSU and targets of novel treatment approaches (e.g., small molecule inhibitors) in an era of personalized medicine.

## Figures and Tables

**Table 1:** Laboratory findings in the examined participants.

Parameters	CSU Group (n = 15)		Control Group (n = 15)		*p*-Value
	$\overset{´}{x}\;\pm \;\mathrm{SD}\;(\mathrm{Range})$	Minimal–Maximal Value	$\overset{´}{x}\;\pm \;\mathrm{SD}\;(\mathrm{Range})$	Minimal–Maximal Value	
VEGF (ng/mL)	410.124 ± 67.174	248.42–533.191	103.523 ± 53.672	69.36–160.528	0.008
PAF (ng/mL)	14.615 ± 4.849	9.171–17.317	14.308 ± 2.205	8.125–18.045	0.999
EDN (ng/mL)	24.978 ± 22.695	6.922–47.155	13.288 ± 2.537	9.777–20.984	0.421

$\overset{´}{x}$-mean; CSU—chronic spontaneous urticaria; SD—standard deviation; VEGF—Vascular Endothelial Growth Factor; PAF—Platelet Activating Factor; EDN—Eosinophil-Derived Neurotoxin.

**Table 2:** Clinical characteristic of the examined patients with chronic spontaneous urticaria.

Parameters	CSU Group (n = 15)		Correlation		
	$\overset{´}{x}\;\pm \;\mathrm{SD}\;(\mathrm{Range})$	Minimal–Maximal Value	VEGF	PAF	EDN
UAS7 scale (points)	31.0 ± 4.95	22–37	r = 0.7 *p* = 0.188	r = 0.3 *p* = 0.624	r = −0.2 *p* = 0.747
Pruritus (VAS) (points)	6.4 ± 1.673	4–8	r = 0.29 *p* = 0.411	r = −0.02 *p* = 0.959	r = 0.1 *p* = 0.784

$\overset{´}{x}$-mean; SD—standard deviation; CSU—chronic spontaneous urticaria; UAS7—The Urticaria Assessment Scale in 7 days; VAS—The Visual Analogue Scale; VEGF—Vascular Endothelial Growth Factor; PAF—Platelet Activating Factor; EDN—Eosinophil-Derived Neurotoxin.

## Data Availability

The data that support the findings of this study are available from the corresponding author upon request.
